# Case report of an arteriovenous graft for renal dialysis, with multiple complications treated successfully over 5 years

**DOI:** 10.1016/j.ijscr.2019.11.059

**Published:** 2019-12-07

**Authors:** T. Mansoor, D. Healy, D. Moneley

**Affiliations:** a Royal College of Surgeons, Ireland; b Beaumont Hospital Dublin, Ireland

**Keywords:** Case report, Arteriovenous graft, Arteriovenous fistula, Fistula stenosis, Fistuloplasty, Graft infection, Dialysis access

## Abstract

**Introduction:**

Arteriovenous grafts (AVG) is a good alternative when native arteriovenous fistula (AVF) is not possible. However, complications are higher and close surveillance is required for successful salvage intervention.

**Presentation of case:**

We present the case of a 35 year old man with a history of a successful Brachio-Axillary AVG performed in his right arm in 2012. He had a background of multiple previous failed attempts of a native AVF formation. He presented in 2014 with symptoms consistent with AVF stenosis. He underwent a successful fistuloplasty and 2 stent insertion. In June 2015 he presented again with re-stenosis and successful fistuloplasty was performed with balloon dilatation. In October 2015 he presented with AVF thrombosis. This was treated with fistuloplasty and thrombolysis. He presented again in August 2016 with a recurrent thrombosis in his AVG and this was again treated with a successful fistuloplasty and thrombolysis on two separate occasions. He then presented in September 2016 with re-stenosis. This was treated with fistuloplasty and 2 stent insertion. He underwent a successful renal transplant during this time and presented again with a sinus discharge in February 2019 when the plan was made for subtotal graft excision.

**Conclusion:**

Incidence of complications is higher when a graft is used over a native AVF. However, close surveillance and prompt intervention can lead to multiple successful salvage procedures thus prolonging the lifespan of the graft. As in our case we were able to prolong the lifespan of the AVG with multiple successful interventions.

## Highlights

• Case report of 35 year old patient with arteriovenous graft formation (AVG).
• Multiple interventions performed with successful salvage over a course of 5 years.
• Outline of role of arteriovenous grafts and current literature and recommendations.
• The learning point from our case is that close monitoring and surveillance can lead to a prolongation of an active AVG.

## 1 Introduction

Much effort has been made in recent times to maximize placement of native arteriovenous fistula (AVF) over grafts with advent of Fistula First Initiative and KDOQI guidelines recommending a native AVF as the vascular access of choice due to superior outcomes [1]. However an arteriovenous graft (AVG) is a good alternative when native AVF is not a viable option, in patients with previous failed attempts, or in the elderly patient with multiple co-morbidities where there is a higher risk of non-maturation [2].

Complication rates are high with a one year and two year primary patency rate ranging between 40–50% and 20–30% respectively, and a one year and two year secondary patency rate ranging between 70–90% and 50–70% often with multiple interventions to maintain patency [3]. A considerable cause for AVG related morbidity is graft stenosis, thrombosis and infection and timely intervention is required to prevent graft failure.

We report a case of an AVG performed in 2012 that sustained multiple complications but with successful monitoring and timely intervention we were able to salvage the AVG for up to 5 years by which time successful renal transplantation was possible. Our case report is in accordance with the SCARE guidelines for reporting cases [4].

## 2 Case presentation

A 35 year old man with end stage renal disease secondary to IgA nephropathy presented for an elective AVG formation in August 2012. He had previously had multiple attempts at a native AVF formation with a failed left brachio-cephalic AVF in 2009 and a failed right brachio-basilic AVF in March 2012. It was decided based on his previous surgeries and small caliber superficial veins that a brachio-axillary polytetraflouroethylene (PTFE) AVG would be the most suitable option for him.

**Fig. 1:**
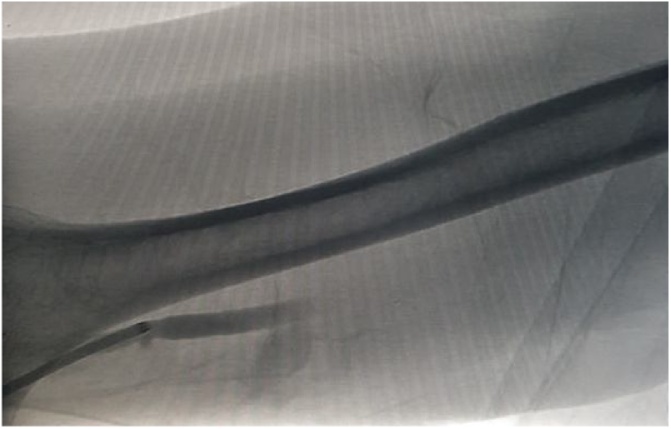
Fistulogram showing occluded graft.

**Fig. 2:**
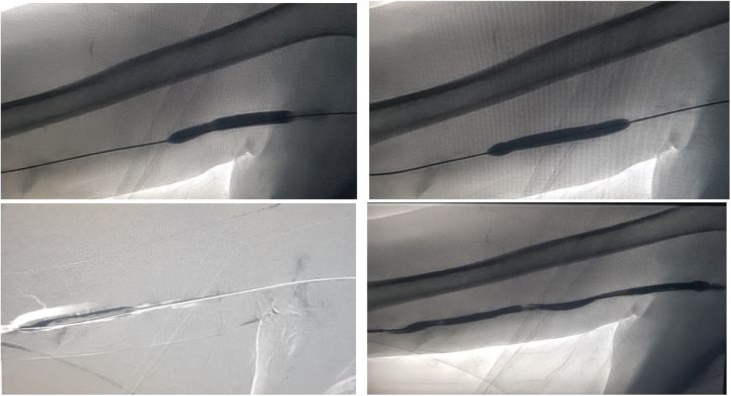
Angioplasty performed in 2014 on occluded segments and a final angiogram showing a good post procedure angiographic outcome.

**Fig. 3:**
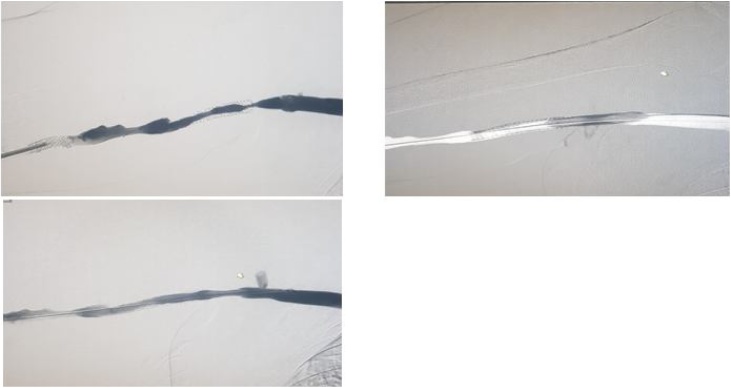
Series of angiograms showing a pre-treatment and post balloon angioplasty graft flow.

He underwent successful surgery and was discharged. In 2014 there was difficulty noted in using the AVG for dialysis. Subsequent imaging confirmed graft stenosis at two different sites. He underwent successful fistuloplasty with interventional radiology with placement of two stents, an 8 mm stent at the proximal graft and a 7 mm stent at the distal graft (Figs. 1–2 ). He again returned in June 2015 with recurrence of stenosis. This was treated successfully with fistuloplasty. However, in October 2015 he had occlusion of his AVG and imaging confirmed a thrombosis. He was treated with fistuloplasty and thrombolysis and discharged with successful treatment. However, in April 2016 he required a further fistuloplasty to treat stenosis (Fig. 3). In August 2016 he developed a further thrombus and this was again treated successfully with thrombolysis. In September 2016 he had a recurrence of his stenosis. This was treated with placement of 2 further stents.

In 2019 he presented with a sinus discharge over the medial aspect of his right upper arm with pain and swelling. By this point he had a successful renal transplant and the AVG was no longer in use for access. On examination a small sinus was noted over the graft site with active purulent discharge and surrounding erythema. He was however systemically well with normal vitals and with only mildly elevated inflammatory markers. An US scan was performed that suggested possible collection in association with the graft and a DVT was ruled out. At this point it was decided to undergo surgical excision of the AVG.

**Fig. 4:**
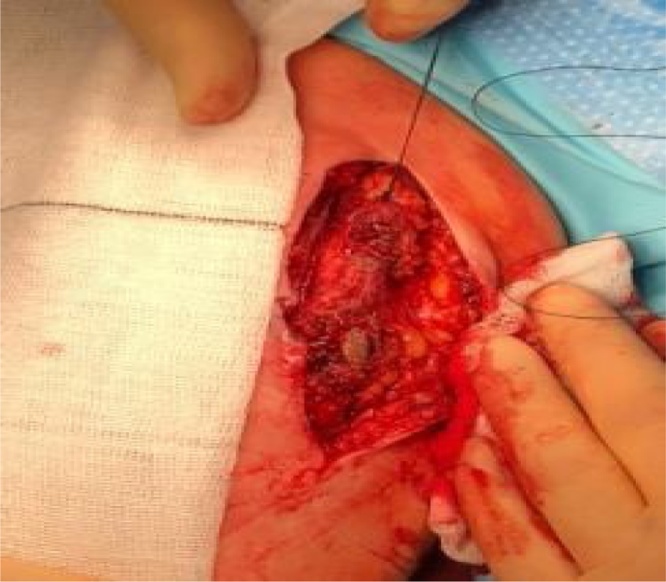
Proximal control obtained. Also seen is sinus tract adjacent to eroded segment of graft.

**Fig. 5:**
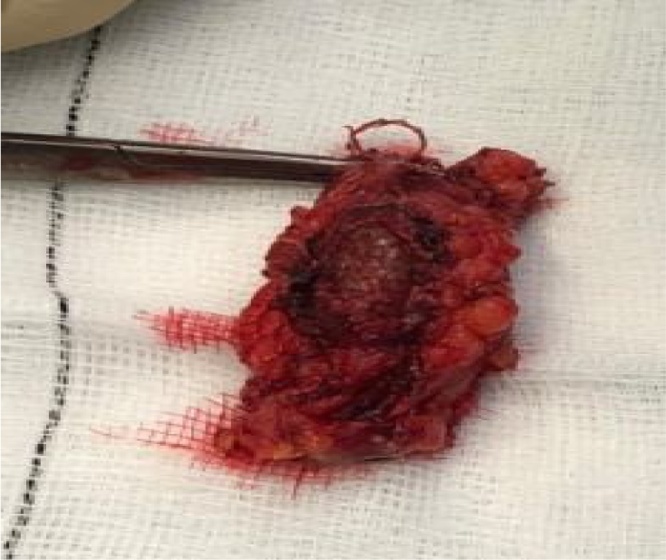
Stent clearly visible through eroded segment of graft.

**Fig. 6:**
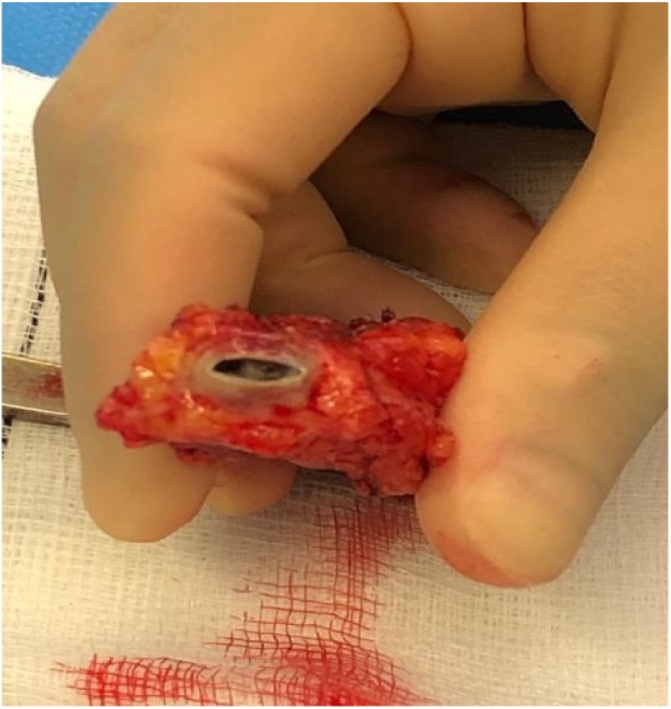
Intra-luminal thrombosis occluding graft in cross sectional view.

Our patient was taken to theatre. Under general anesthetic a skin incision was made to gain proximal and distal control before a dissection was made into the graft. A note was made of an eroded graft wall with a 1 × 2 cm defect revealing an underlying stent device and intra-luminal thrombosis (Figs. 4–6 ).

The patient recovered well and was subsequently discharged post-operative day 1 with a course of oral antibiotic and for OPD follow up.

## 3 Discussion

This case demonstrates that although complications are common with a AVG close surveillance and timely intervention can prolong access lifespan. In this case, we were well aware of the difficulty that a failed AVG would pose for this gentleman, given his extensive history of failed AVF formation and co-morbidities. Therefore, stringent monitoring was employed with rapid intervention to treat the various complications that presented over the course of 7 years.

AVF infection is an important consideration with incidence ranging between 4–20% with the use of a graft [5]. Multiple different factors predispose patients to getting a graft infection. Uremia related immunodeficiency due to ESRD, multiple co-morbidities, and vascular access technique during time of hemodialysis are all thought to be associated factors. Diagnosis is clinical with local signs such as redness, swelling, warmth, pain, discharge. Systemic symptoms may indicate sepsis and requires urgent attention. US imaging may be used to aid diagnosis by looking for fluid collections. The ESVS guideline recommendation for graft infection is total graft infection if sepsis is present. Partial excision may be considered if segments of graft appear intact [3] as was the case with our patient.

Stenosis can occur anywhere along the site of graft but often occurs in the juxta-anastomotic areas. Assessment for stenosis can be made in a physical exam. A change in the thrill or pulsatile flow can indicate stenosis. Reduced flow during hemodialysis may also be seen. The recommendation for treatment is percutaneous transluminal angioplasty (PTA) if inflow or outflow stenosis is suspected [3]. Stenosis can lead to abnormal or reduced flow which can then increase the risk of thrombosis and therefore pre-emptive treatment may be considered in select cases [6].

Graft thrombosis often presents secondary to progressive stenosis. Early treatment of thrombosis is recommended to prevent organization of thrombus. Treatment options include thrombectomy and thrombolysis however this is not sufficient as a flow limiting stenosis will also need to be treated to ensure improved patency. Endovascular therapy was shown to have similar patency rates to surgical thrombectomy [7]. The ESVS guidelines recommend either surgery or endovascular approach depending on centre expertise provided there is concomitant treatment of any associated stenosis [3].

An important aspect to discuss is the role of surveillance and patient/ staff education in monitoring for graft complications. Patients should be educated in examining their AVG and in identifying any abnormal patterns or signs. Any concerns should be flagged with the hemodialysis unit and the primary team. Examination should also take place during visits for hemodialysis and any concerns for complications should be investigated with further imaging. Monitoring is shown to be cost-effective method in improving patency [8].

In our case, we were fully aware of the challenges associated with a failed graft and our patient was young and fully compliant with education and surveillance measures. We were able to ensure timely interventions that allowed for successful salvage of his AVG until renal transplant was possible.

## 4 Conclusion

Complication rates are higher in a AVG with lower patency rates when compared to native AVF. However, close surveillance and prompt intervention can lead to multiple successful salvage procedures thus effectively prolonging the life of the AVG. As in our case we were able to prolong the life of the AVG with 6 successful interventions.

There may be role for an individualized approach in managing patients with AVG. Patient education and staff education in recognizing early signs of complications with tailored surveillance programs can help in optimizing outcome.

## Sources of funding

Case report not sponsored.

## Ethical approval

Not applicable as no research on human participants was involved.

## Consent

Written informed consent was obtained from the patient for publication of this case report and accompanying images. A copy of the written consent is available for review by the Editor-in-Chief of this journal on request.

## Author contribution

Tayyaub Mansoor 1st author - Conceptualisation, Methodology, Resources, Writing.

Donagh Healy 2nd author - Supervision.

Darragh Moneley 3rd author - Supervision.

## Registration of research studies

Not applicable as this manuscript does not involve research on human participants.

## Guarantor

Darragh Moneley.

Beaumont Hospital.

Dublin Ireland.

dmoneley@gmail.com.

## Provenance and peer review

Not commissioned, externally peer-reviewed.

## Declaration of Competing Interest

No conflict of interest.

## References

[1] Besarab A., Ford Hospital H, Work J, Brouwer D., McMurray C., Timothy Bunchman P.E. (2006). https://www.ajkd.org/article/S0272-6386(06)00646-9/pdf.

[2] Lee H.W., Allon M. (2013). When should a patient receive an arteriovenous graft rather than a fistula?. Semin. Dial..

[3] Schmidli J., Widmer M.K., Basile C., de Donato G., Gallieni M., Gibbons C.P. (2018). Editor’s choice – vascular access: 2018 clinical practice guidelines of the european society for vascular surgery (ESVS). Eur. J. Vasc. Endovasc. Surg..

[4] Agha R.A., Borrelli M.R., Farwana R., Koshy K., Fowler A.J., Orgill D.P. (2018). The SCARE 2018 statement: updating consensus Surgical CAse REport (SCARE) guidelines. Int. J. Surg..

[5] Padberg F.T., Calligaro K.D., Sidawy A.N. (2008). Complications of arteriovenous hemodialysis access: recognition and management. J. Vasc. Surg..

[6] Dember L.M., Holmberg E.F., Kaufman J.S. (2004). Randomized controlled trial of prophylactic repair of hemodialysis arteriovenous graft stenosis. Kidney Int..

[7] Kuhan G., Antoniou G.A., Nikam M., Mitra S., Farquharson F., Brittenden J. (2013). A meta-analysis of randomized trials comparing surgery versus endovascular therapy for thrombosed arteriovenous fistulas and grafts in hemodialysis. Cardiovasc. Intervent. Radiol..

[8] Coentrão L., Faria B., Pestana M. (2012). Physical examination of dysfunctional arteriovenous fistulae by non-interventionalists: a skill worth teaching. Nephrol. Dial. Transplant..

